# Accuracy of PET quantification in [^68^Ga]Ga-pentixafor PET/MR imaging of carotid plaques

**DOI:** 10.1007/s12350-020-02257-3

**Published:** 2020-07-21

**Authors:** Ivo Rausch, Dietrich Beitzke, Xiang. Li, Sahra Pfaff, Sazan Rasul, Alexander R. Haug, Marius E. Mayerhoefer, Marcus Hacker, Thomas Beyer, Jacobo Cal-González

**Affiliations:** 1 QIMP Team, Center for Medical Physics and Biomedical Engineering, Medical University of Vienna, Waehringer Guertel 18-20, 1090 Vienna, Austria; 2 Division of Cardiovascular and Interventional Radiology, Department of Biomedical Imaging and Image-guided Therapy, Medical University of Vienna, Vienna, Austria; 3 Division of Nuclear Medicine, Department of Biomedical Imaging and Image-guided Therapy, Medical University of Vienna, Vienna, Austria; 4 Christian Doppler Lab for Applied Metabolomics, Medical University of Vienna, Vienna, Austria; 5 Division of General and Pediatric Radiology, Department of Biomedical Imaging and Image-guided Therapy, Medical University of Vienna, Vienna, Austria

## Abstract

**Aim:**

The aim of this study was to evaluate and correct for partial-volume-effects (PVE) on [^68^Ga]Ga-Pentixafor uptake in atherosclerotic plaques of the carotid arteries, and the impact of ignoring bone in MR-based attenuation correction (MR-AC).

**Methods:**

Twenty [^68^Ga]Ga-pentixafor PET/MR examinations including a high-resolution T2-TSE MR of the neck were included in this study. Carotid plaques located at the carotid bifurcation were delineated and the anatomical information was used for partial-volume-correction (PVC). Mean and max tissue-to-background ratios (TBR) of the [^68^Ga]Ga-Pentixafor uptake were compared for standard and PVC-PET images. A potential influence of ignoring bone in MR-AC was assessed in a subset of the data reconstructed after incorporating bone into MR-AC and a subsequent comparison of standardized-uptake values (SUV).

**Results:**

In total, 34 atherosclerotic plaques were identified. Following PVC, mean and max TBR increased by 77 and 95%, respectively, when averaged across lesions. When accounting for bone in the MR-AC, SUV of plaque changed by 0.5%.

**Conclusion:**

Quantitative readings of [^68^Ga]Ga-pentixafor uptake in plaques are strongly affected by PVE, which can be reduced by PVC. Including bone information into the MR-AC yielded no clinically relevant effect on tracer quantification.

**Electronic supplementary material:**

The online version of this article (10.1007/s12350-020-02257-3) contains supplementary material, which is available to authorized users.

## Introduction

Ischemic stroke is one of the main causes of death or severe permanent disability worldwide.^1^ Stroke events are often caused by the disruption of unstable plaques in the carotid arteries, which leads to the interruption of blood flow to the brain, and, therefore, to damage or death of brain cells. Often referred to as *vulnerable plaques*, these unstable plaques have characteristics, such as increased inflammatory cell infiltration, large lipid cores, or thin fibrous caps.^2^,^3^

Several studies demonstrated that [^18^F]FDG-PET imaging is a reliable non-invasive approach to assess inflammation in atherosclerotic plaques.^4^ [^18^F]FDG uptake correlates well with the rate of cardiovascular events.^5^ However, several limiting factors compromise the use of [^18^F]FDG for imaging inflammation of atherosclerotic plaques,^6^ such as (1) spill-in effects from nearby tissue with physiological uptake; (2) the influence of blood glucose levels on [^18^F]FDG uptake; (3) the presence of brown adipose tissue with increased glycolytic activity; and (4) the need for a long fasting period or dedicated diet before imaging, which compromise patient comfort. Therefore, specific radiotracers that target inflammatory cells are welcome to better characterize atherosclerotic plaques.

The role of the CXCR4 chemokine receptor and its ligand CXCL12 in atherosclerosis was recently described.^7^–^10^ The CXCR4/CXCL12 complex plays an important role in the hematopoiesis in bone marrow,^11^ apoptosis, and pro-inflammatory progression in atherosclerotic plaques.^12^ Due to the over-expression of CXCR4 at the immune-cells involved in the inflammatory process, [^68^Ga]Ga-Pentixafor, a radiotracer with high affinity to CXCR4, has been suggested as a imaging marker of inflammatory cells with PET imaging.^13^

Both inflammatory processes and plaque structure can be assessed within a single examination using PET/MRI and [^68^Ga]Ga-Pentixafor as the tracer-of-choice. For example, Li et al. evaluated the reproducibility of [^68^Ga]Ga-Pentixafor uptake quantification of atherosclerotic plaques with PET/MRI and the relation between CXCR4 expression and the cardiovascular risk profile of the patients, and demonstrated significantly increased [^68^Ga]Ga-Pentixafor uptake in patients with cardiovascular risk profiles.^14^ Nonetheless, the quantification accuracy of [^68^Ga]Ga-Pentixafor uptake in plaques was limited.

General limitations of PET quantification notwithstanding,^15^,^16^ quantitative evaluation of plaques using [^68^Ga]Ga-Pentixafor PET/MRI is hampered by two factors: the lack of bone information during MR-based attenuation correction (AC),^17^ and partial-volume-effects (PVE) that arise from the size of the vulnerable plaques being comparable to that of the PET detector elements.^18^ These PVE are of particular interest in ^68^Ga-based PET examinations since the mean positron range inherent to the decay of gallium-68 is about 3-4 mm. This implies an additional blurring of the images contributing to a further loss of spatial resolution.^19^ Therefore, partial-volume-correction (PVC) is mandatory for a proper quantification of PET uptake in atherosclerotic plaques.^20^

The aim of this work was to evaluate the impact of both, PVC and ignoring bone in MR-based AC on [^68^Ga]Ga-Pentixafor uptake measures in atherosclerotic plaques in the carotid arteries. We employ a recently validated method for PVC^21^,^22^ for quantification of atherosclerotic plaques in the proximity of the carotid bifurcation. Furthermore, a subset of datasets was reprocessed adding bone in the MR-AC to assess the influence of bone during MR-AC on quantification, and quantitative readings were compared to the standard MR-AC PET.

## Materials and Methods

### Patient Population

Eighteen lymphoma patients (10 male, 8 female, mean age: (64 ± 10) years, range 47 to 79 years) who presented with an arterial focal uptake on [^68^Ga]Ga-Pentixafor PET/MR images with a corresponding anatomical alteration were assessed retrospectively. Two patients also underwent a follow-up [^68^Ga]Ga-Pentixafor PET/MR scans 119 and 90 days after the first scan. All scans were performed between October 2016 and May 2017. In total, 20 datasets were evaluated.

All patient data originated from a prospective study investigating the CXCR4 expression in patients with mucosa-associated lymphoid tissue (MALT) lymphoma. The study was approved by the Institutional Ethics Committee and was in accordance with the 1964 Helsinki declaration and its later amendments; all patients provided written informed consent. Parts of the data used in this study were also reported elsewhere.^23^,^24^

### PET/MRI

All scans were performed on a Biograph mMR PET/MR system (Siemens Healthineers GmbH).^25^ The patients were injected with (156 ± 11) MBq of [68 Ga]-Pentixafor and underwent a whole-body PET/MR examination using a scan time of 5 minutes per bed position (98 ± 13) min post injection. A 3D-Ordinary Poisson Ordered-Subsets-Expectation-Maximization (OP-OSEM) algorithm, with point-spread-function (PSF) correction using three iterations and 21 subsets, was used for PET image reconstruction. Attenuation correction was performed using the standard DIXON-based MR-AC implemented in the PET/MRI system (Software version VB20). A single bed position image centered in the head-neck region, with 344 × 344 × 127 matrix size and 2.0863 × 2.0863 × 2.031 mm^3^ voxel size, was evaluated for each scan. The images were smoothed with a 4-mm full-width at half-maximum (FWHM) Gaussian filter.

### Plaque Segmentation and Classification

**Figure 1:**
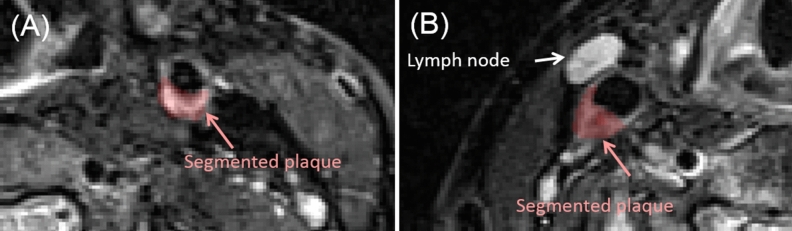
Examples of plaque delineations. (**A**) Vulnerable atherosclerotic plaques were identified and segmented on T2-TSE MR images. (**B**) Lymph nodes with non-negligible [^68^Ga]Ga-Pentixafor uptake located close to the segmented plaques were also included in the PVC evaluation

T2-weighted Turbo Spin Echo (TSE) magnetic resonance (MR) images (256 × 256 × 14 voxels and 0.625 × 0.625 × 2.2 mm^3^ per voxel) co-registered to the PET images were used for the delineation of the plaques. All the identified atherosclerotic plaques were manually delineated on T2-TSE MR images (Figure 1) by an experienced radiologist (DB, 10-y experience in vascular imaging). [^68^Ga]Ga-Pentixafor-positive lymph nodes, located close to the segmented plaques, were also segmented and included in the PVC evaluations. A threshold-based segmentation of the T2-TSE MR image was used for segmenting the lymph nodes located close the plaques.

The segmented plaques were classified into five different types, with regard to the plaque morphology observed in the T2-TSE MR image. The classification was based on the modified American Heart Association (AHA) of atherosclerotic Plaque for MRI^26^ (Table 1).

**Table 1:** Plaque classification according to the MRI morphology

Classification	MR morphology	Corresponding modified AHA Classification for MRI* (ref)
Type 1	Uniform plaque morphology. Regular borders	Type I–II: near-normal wall thickness, no calcification
Type 2	Atherosclerotic plaque with intermediate eccentric thickening, no calcification	Type III: diffuse intimal thickening of small eccentric plaque with no calcification
Type 3	Relatively large plaque. Fibrous tissue with lipid-rich necrotic core. Regular borders	Type IV–V: plaque with a lipid or necrotic core surrounded by fibrous tissue with possible calcification
Type 4	Very irregular morphology and borders	Type VI: complex plaque with possible surface defect, hemorrhage, or thrombus
Type 5	Advanced plaque with the presence of calcifications and regular borders	Type VII: calcified plaque

*The plaque classification was based on the modified AHA classification of atherosclerotic plaque.^26^ However, the classification in this study was performed only using the T2-TSE MRI, and thus, cannot be regarded as fully equivalent

### Partial Volume Correction

The local projection (LP) method was used for the PVC of the segmented plaques.^21^,^22^ This method is applied locally, using a small volume-of-interest (VOI) that includes the target lesion. The method models the measured counts in the projection space as the sum of the counts from each segmented tissue, scaled by their respective tracer uptake, plus the counts originated from the image outside the local VOI where the lesion of interest is included. This model is illustrated by Eq. (1):

1 $$\lambda _{i} \equiv \mathop \sum \limits_{{j = 1}}^{J} A_{j} P_{{ij}} + g_{{{\text{out}},i}}$$

where *λ*_*i*_ are the expected counts per sinogram bin, i, *A*_*j*_ the activity for each segmented tissue j, *P*_*ij*_ is the resolution-blurred tissue shape function for tissue *j*, and sinogram bin *i* and $g_{{{\text{out}},i}}$ represents the background counts coming from the region outside the segmented tissues in each sinogram bin *i*. The tissue activities *A*_*j*_ (LP tissue activities) are determined by fitting the measured projection data to the model in Eq. (1) and are used as a prior in a new reconstruction procedure. Of note, the prior corresponds to the theoretical calculated activity during the LP method. The calculated priors expressed at TBRs can be found in Supplement Table 1. This step leads to a locally applied PVC and a locally corrected PET image with improved visual appearance and quantification of the lesions of interest. Further details on the method are provided in Ref. 22.

In order to adjust (*P*_*ij*_) to the expected clinical resolution of the system using the applied reconstruction settings for 68 Ga, the system resolution was modeled from experimental scans of the NEMA-NU2 Image Quality phantom. Here, the FWHM of the Gaussian blurring was obtained by fitting the LP tissue activities for the 37 mm sphere of the NEMA IQ phantom, filled with an aqueous solution containing [68 Ga] and reconstructed following the same settings as used in the patient studies to the theoretical activities which were used in the phantom experiment.^22^

### Data Analysis

The volume of the segmented plaque was calculated from the T2-TSE MR images by summing all voxels within the plaque. The figure-of-merit for PET was the target-to-background ratio (TBR) using the max (TBR_max_) and mean (TBR_mean_) pixel value within the segmented plaque. Here, the background region was measured in the blood pool. The blood pool was defined by means of a VOI (494 ± 30 mm^3^) in a venous region located far from any plaque and/or lymph node to avoid spill-in contaminations.

The quantitative impact of PVC was evaluated by means of the relative change in the TBR (ΔTBR) of the segmented plaque after applying the PVC:

2 $$ \Delta {\text{TBR}} \left( {\text{PVC}} \right) = \frac{{{\text{TBR}} \left( {\text{PVC}} \right) - {\text{TBR}}\left( {\text{noPVC}} \right)}}{{{\text{TBR}}\left( {\text{noPVC}} \right)}} \cdot 100  \left( \% \right), $$

where TBR(PVC) is the target-to-background ratio after applying the PVC (measured from the tissue activities obtained with the LP method or from the PVC image) and TBR(noPVC) is the lesion-to-background ratio measured in the image without PVC.

### Statistical Analysis

Preliminary remark: The choice of the statistical tests to analyze the data is based on the assumption that the tracer uptake, and thus, the TBRs in the individual plaque groups are normal distributed. Consequently, the pooled data are assumed to be a distribution that corresponds to the sum of five normal distributions with different mean values, weighted by their ratio of included samples. Thus, for analysis of the combined data no normal distribution is expected, and therefore, non-parametric statistical tests were used. Further, we assumed no dependence between plaques in the right and left carotid artery within an individual patient, and thus, these plaques were treated as individual measurements.

A paired sample Wilcoxon signed-rank test was used to test for statistically significance of all combined TBR changes after PVC. To test for statistical significance of the TBR changes after PVC in the individual groups, a paired student’s *t* test was used. To test if PVC changes significantly the discriminability between [Ga68]Ga-Pentixafor uptake between the plaque types, an ANOVA followed by a Tukey’s HSD test was used. Further, correlation analysis was performed between quantitative measures using the Pearson’s correlation method and between quantitative measures and ordinal variables using polyserial correlation. For all statistical tests, a *p* value of *p* < 0.05 was considered as significant. All statistical calculations were done using the software *R: A language and Environment for Statistical computing* (R Foundation for Statistical Computing, Vienna, Austria).

### Evaluation of the Impact of Ignoring Bone in MR-Based AC

The effect of ignoring bone tissue on standard MR-AC (MR-AC_std_) was investigated in a subset of the first 11/18 patients. Here, spatially variant attenuation coefficients of the skull and the cervical spine were added to the standard DIXON-based AC method. This was done using a non-commercial prototype version of the five compartment AC method available for the mMR PET/MRI system in software version E11.^27^ Then, the PET raw data were reconstructed again using the model-based MR-AC (MR-AC_bone_) applying the same image reconstruction parameters as described above. The plaque delineations were copied to the respective PET images reconstructed with and without accounting for bone in the MR-AC, and maximum and mean SUV values were extracted. The contribution of bone on the quantification was evaluated by means of relative differences in SUV (ΔSUV):

3 $$ \Delta {\text{SUV}} \left( {\text{AC}} \right) = \frac{{{\text{SUV}} \left( {{\text{MR}} - {\text{ACbone}}} \right) - {\text{SUV}}\left( {{\text{MR}} - {\text{ACstd}} } \right)}}{{{\text{SUV}}\left( {{\text{MR}} - {\text{ACstd}}} \right)}} \cdot 100   \left( \% \right) $$

A paired sample Wilcoxon signed-rank test was used to evaluate statistically significant changes in the ΔSUV_max_ and ΔSUV_mean_.

## Results

**Figure 2:**
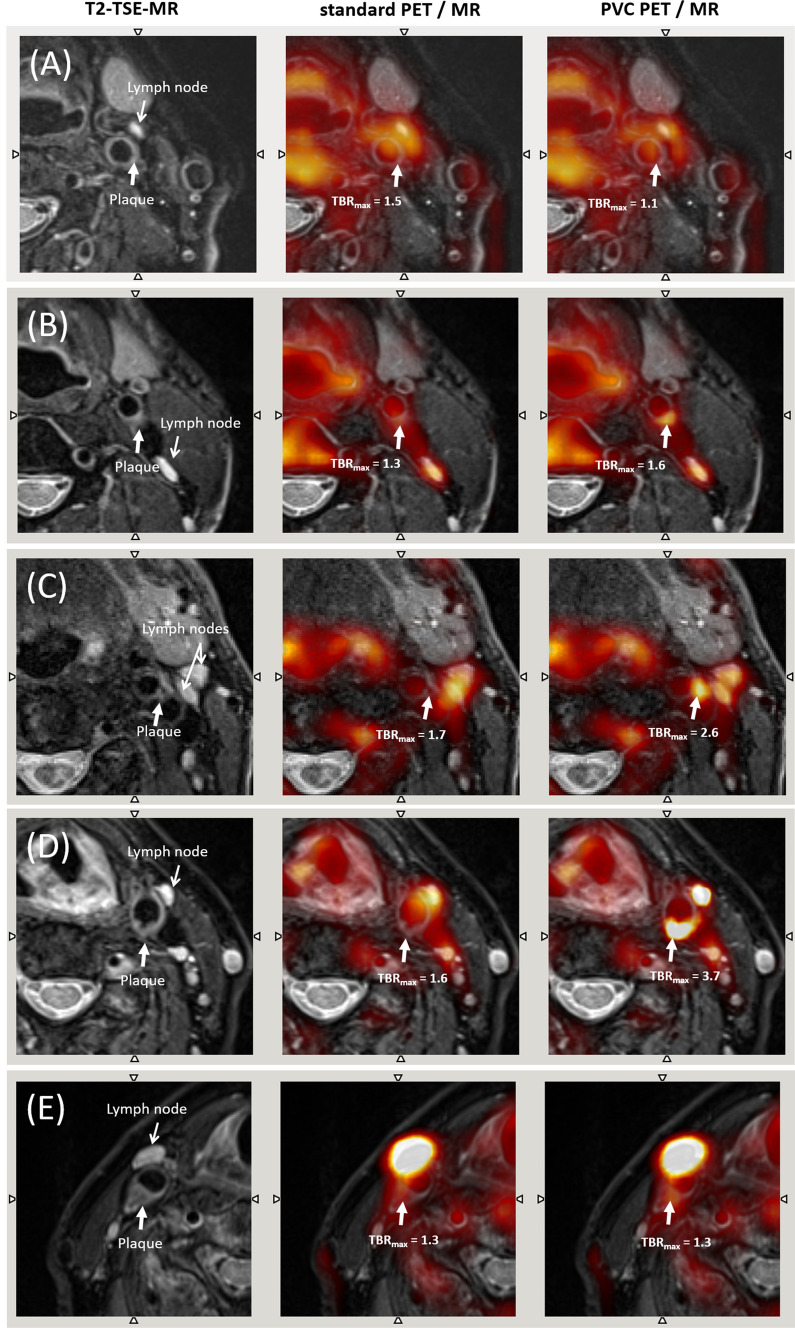
Axial MR (left) and PET/MR images before (centre) and after PVC (right) of different plaque types. From top to bottom (**A**-**E**) examples of plaques of type 1 to 5, respectively, are shown. (**A**) For plaque type 1, a small plaque with uniform morphology can be seen in the wall of the left carotid on T2-TSE MR. Unexpectedly for this type of plaque, a minor increase in uptake can be seen on the non-PVC-PET image (TBR = 1.5). However, when correcting the PVE, the TBR of the plaque is reduced to 1.1. This can be explained by the spill-in effects from a nearby hypermetabolic lymph node as seen on non-PVC already. As expected, for plaque types 2, 3, and 4 (**B**, **C**, and **D**), the PVC recovers the uptake in the plaque, thus, resulting in significantly increased TBR’s. (**E**) For plaque type 5, the PVC did not have a significant effect in the measured uptake of the plaque, due to the large size of the plaque. A small spill-in effect from a nearby lymph node is appreciated, but without an appreciable effect in the TBR values

Thirty four atherosclerotic plaques were identified and segmented from the T2-TSE MR images in all scans. Of those, two were classified based on T2-TSE MRI as plaque (type 1), 12 as plaque (type 2), 13 as plaque (type 3), two as plaques (type 4), and 5 as plaque (type 5). Figure 2 shows the T2-TSE MR image for a representative case of each plaque type. A table with the classification of all individual plaques together with all measured TBR values is given in Supplement Table 1.

### Influence of Partial Volume Correction

Figure 2 shows the original and the PVC image for a representative case of each plaque type. For plaque types 1 and 5, the PVC did not change the visual image appearance of the plaque on PET. However, for plaques categorized as type 2, 3, and 4, a substantial increase in tracer uptake readings was observed. Furthermore, after PVC, the visual appearance of the activity distribution in the plaques as well as for the [^68^Ga]Ga-Pentixafor positive lymph nodes was less blurred and presented with an improved alignment with the underlying anatomical structures.

**Figure 3:**
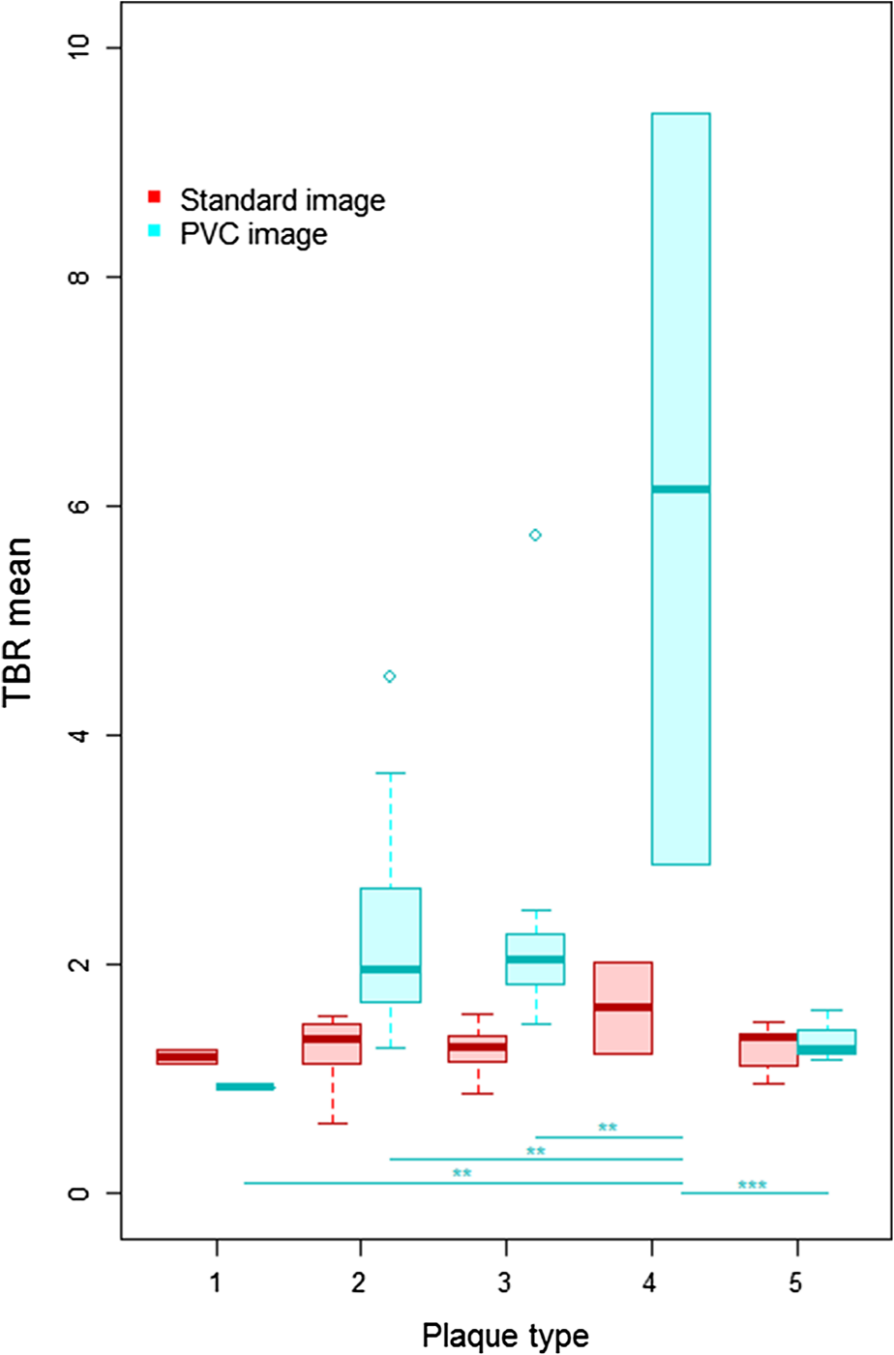
Boxplots of the TBR mean values of the different plaque types extracted from the standard PET and the PVC-PET. Statistically significant differences of TBR means before and after PVC were only present for plaque type 1, 2, and 3. Boxes depict the interquartile range (25% to 75%) with the bold line showing the median. Whiskers depict the minimum and maximum value and open points depict outliers (> 1.5 × the IQR away from the nearest quartile). Statistical significance between groups is encoded in the figure as *p* < 0.05 (*), *p* < 0.01 (**) and *p* < 0.001 (***)

**Figure 4:**
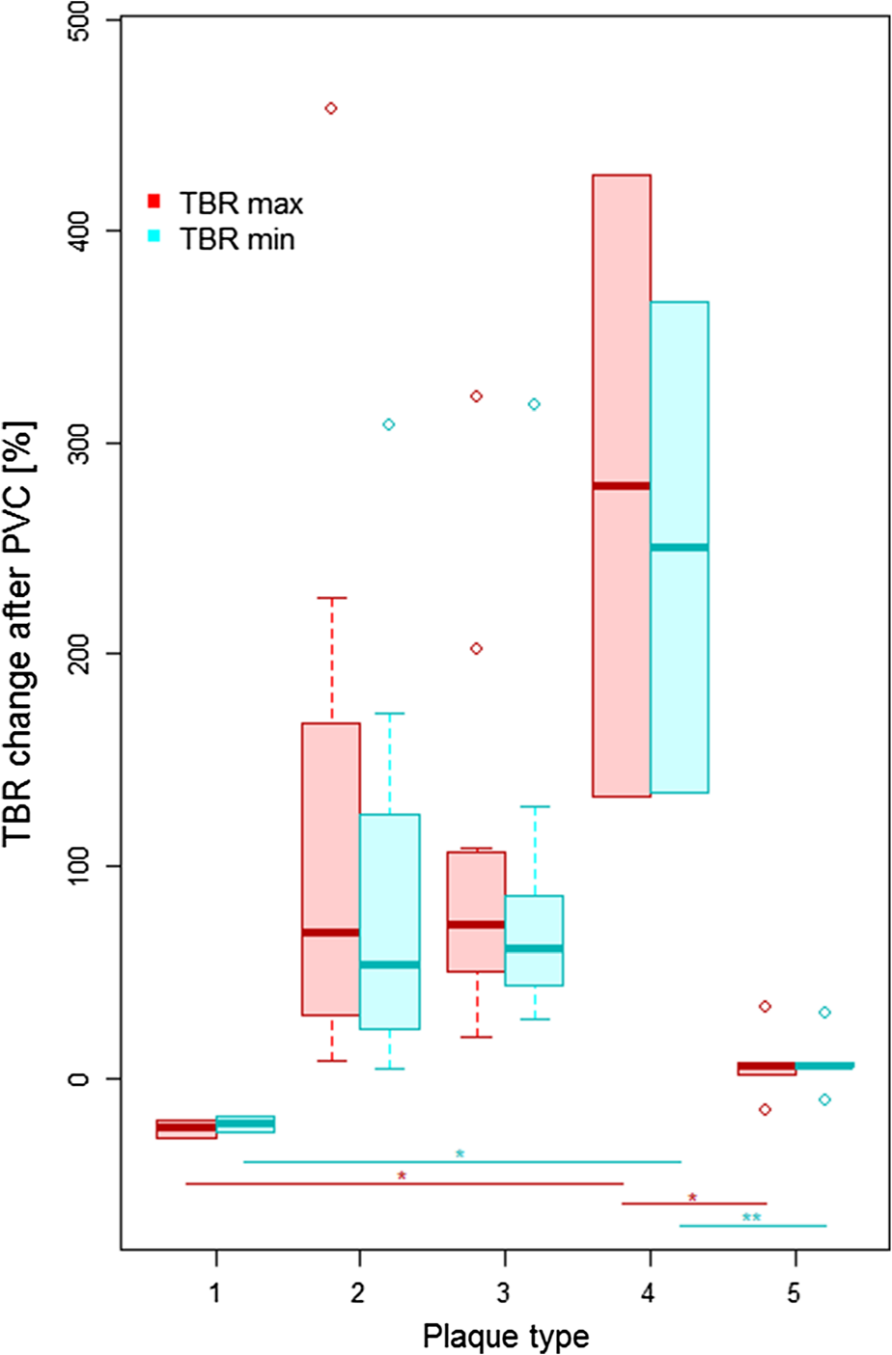
Boxplots of the relative differences (ΔTBRs) between the maximum and mean TBRs from standard PET and the PVC-PET for all different plaque types. Boxes depict the interquartile range (25-75%) with the bold line showing the median. Whiskers depict the minimum and maximum value and open points depict outliers (> 1.5 × the IQR away from the nearest quartile). Statistical significance between groups is encoded as *p* < 0.05 (*), *p* < 0.01 (**) and *p* < 0.001 (***)

On average, PVC increased TBR_max_ and TBR_mean_ by 95% (*p* < 0.001) and 77% (*p* < 0.001), respectively, in all plaque lesions. PVC reduced the TBR for plaque lesions of type 1 (ΔTBR_max_ = − 24%, *p* = 0.03; ΔTBR_mean_ = − 22%, *p* = 0.13), but did affect the TBR of lesions of type 5 only minimally (ΔTBR_max_ = 6%, *p* = 0.61; ΔTBR_mean_ = 7%, *p* = 0.36). For lesions classified as type 2 and type 3, TBR (max and mean) was approximately doubled post PVC (all *p* < 0.01). For lesion of type 4, an average increase of the TBR of ΔTBR_max_ = 280% (*p* = 0.36) and ΔTBR_mean_ = 250% (*p* = 0.36) was found. Table 2 summarizes all TBR_max_ and TBR_mean_ values as well as relative differences for all image reconstructions and following the corrections for PVE. Figures 3 and 4 show boxplots of the TBRs and the ΔTBRs that demonstrate the influence of PVC for the different plaque types. All calculated TBRs for the individual plaques are provided in Supplement Table 1.

**Table 2:**
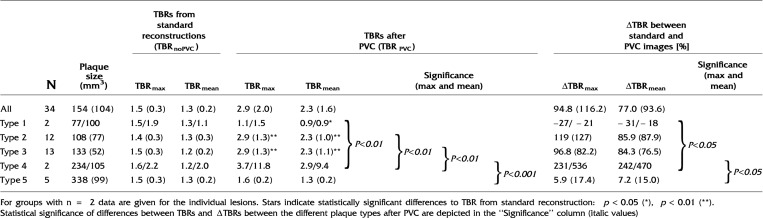
Average plaque size (standard deviation) as well as the TBRs (standard deviation) obtained from the standard and PVC images together with the relative differences in TBR (standard deviation) in [%] for all plaque lesions and plaque types

There were no statistically significant differences between TBR of the different plaque types prior to PVC. Following PVC, statistical differences were found only between TBRs (max and mean) of plaques of type 4 and the TBRs of all other plaque types. Similarly, the ΔTBR (max and mean) showed only significant differences between plaque type 4 and plaque type 1 and 5. No correlation between plaque size and ΔTBR was found, neither for all plaques nor within the individual groups. The only correlation was between plaque type and plaque size (polyserial correlation coefficient: ϱ = 0.72 (*p* < 0.001), p value for testing bivariate normality: *p* = 0.193).

### Impact of Ignoring Bone in the MR-Based AC

In total, 22 plaque lesions were evaluated in 11/18 patients. On average, a statistically significant underestimation of ΔSUV_max_ = (0.5 ± 0.6)% (*p* < 0.001) and ΔSUV_mean_ = (0.4 ± 0.6)% (*p* < 0.001) was found.

## Discussion

In this study, we assessed the effect of PVC and a modified MR-AC on quantification of [^68^Ga]Ga-Pentixafor-PET imaging of atherosclerotic plaque lesions using PET/MRI. Following PVC, the overall image contrast of plaque lesions was improved (TBR increased) for plaque types 2, 3, and 4. For lesions of type 1 and 5, PVC did only minimally affect the TBR values. In some cases, the measured TBR was even reduced due to the correction of spill-in effects that arise from lymph nodes with high uptake located close to the plaque (Figure 2A and E).

Our findings are in line with prior studies evaluating PVE in atherosclerotic plaques imaged using ^18^F-labeled radiotracers.^20^,^22^ For example, in a simulation study by Huet et al., reductions in SUV readings of up to 10% of the actual activity were observed in atherosclerotic plaques.^20^ These reductions are explained by the small size of the plaques, which are often below the spatial resolution of the PET system. However, in case of ^68^Ga-labeled tracers, stronger PVE effects are expected. The decay of gallium-68 inherently transfers a higher average energy to the positron than in the case of the decay of fluorine-18, thus, resulting in a significantly extended positron range.^19^ This leads to a further blurring of the image, and thus, to stronger PVE causing spill-out as well as spill-in effects.

The strong influence of PVE in [^68^Ga]Ga-Pentixafor positive plaque lesions can be seen in the low difference of TBR readings between lesions, when reconstructed without using any PVC method (Table 2). These findings are also in agreement with a study published by Li et al.,^23^ who showed minor, yet significant differences between [^68^Ga]Ga-Pentixafor uptake of differently classified plaque lesions in a similar patient cohort. Given these minor differences in TBRs it seems unlikely that a quantitative evaluation of [^68^Ga]Ga-Pentixafor uptake based on SUV or TBR can be used to classify plaque lesions on an individual patient level.

In order to enable a classification of plaque lesions or to use quantitative [68 Ga]Ga-Pentixafor measures for therapy response, a clinically viable PVC approach is required. As shown in this study, PVC substantially increased individual TBRs specifically in plaques of type 2, 3, and 4 (Figure 3). Though, a general group wise statistical significance could not be shown, these findings seem reasonable given the chosen classification of the plaque lesions. Plaque type 1 and 2 are associated with a very early stage of the plaque development, and therefore, were not expected to present substantial inflammation associated with [68 Ga]Ga-Pentixafor uptake.^28^ However, since type 2 plaques are regarded as the transition between plaque type 1 and 3, an increased [68 Ga]Ga-Pentixafor uptake compared to type 1 plaque lesions might be reasonable. In contrast, plaque type 3 and 4 presented morphologically with lipid cores. Such lipid accumulations are associated with inflammation,^29^ and thus, can be expected to show elevated [68 Ga]Ga-Pentixafor uptake. In contrast, plaque types 5 presented calcified cores. Plaque with macro-calcifications have been shown to present with downregulated markers for macrophages and repressed inflammation.^30^ Hence, a reduced [68 Ga]Ga-Pentixafor uptake compared to plaque types 3 and 4 seems reasonable. These findings are also in agreement with a study on [18F]FDG uptake in atherosclerotic plaques by Hyafil et al.^31^ Here, a significantly higher [18F]FDG accumulation associated with inflammatory processes in plaque type VI (AHA classification) and elevated, though non-significant, [18F]FDG accumulation in plaque type IV-V (AHA classification) was shown, which correspond to plaque type 4 and 3 in this study, respectively.

In general, the ability to assess the absolute activity concentration within the plaques is questionable even after PVC. As shown in a previous study evaluating the used PVC in phantom scans using fluorine-18 only 30% of the actual activity concentration could be recovered for lesions with a diameter of a few millimeters.^22^ Taking into account the findings by Huet et al.,^20^ who demonstrated reductions of activity concentration of up to 95% for small lesions, a much higher change in TBR as found in this study would be expected to fully recover the actual [^68^Ga]Ga-Pentixafor uptake. This behavior is mainly attributed to a non-satisfied Nyquist sampling condition (lesion size > 3 voxels), where the LP PVC method has shown to underestimate the real activity.^32^ Furthermore, the LP PVC assumes a uniform activity in the plaque lesions. However, this assumption may not be correct in clinical practice since a highly localized tracer uptake within a lesion could also affect the performance of the used PVC. Therefore, the quantitative values after PVC cannot be expected to reflect correctly the actual local activity concentration within the plaque lesion. This issue may be overcome by a refinement of the LP method by incorporating a model assuming a non-uniform tracer uptake in the delineated VOI similar as done by Southekal et al. for PVE in SPECT examinations.^33^ Finally, OSEM (with and without PSF correction) may not fully converge for small structures with low TBRs, such as plaque lesions.^34^,^35^ This potentially contributes an additional bias to activity measurements in the plaque lesions, irrespective of the use of a PVC.

Nonetheless, an absolute activity concentration is likely not needed for the use of quantitative measures as SUV or TBR for the classification of inflammatory processes within atherosclerotic plaques using [68 Ga]Ga-Pentixafor. A robust PVC method that is able to reliably and reproducibly recover at least a part of the actual tracer uptake would cause a systematic error but could be used to elevate the difference of quantitative measures, and thus, enable a classification of plaque lesions or the assessment of therapy response on the bases of objective measures.

Finally, only a negligible difference in quantification of plaque lesions in the carotid bifurcation was found for variations of the MR-attenuation template. Prior studies on the effect of adding bone attenuation to this template reported an adjustment of ~ 4% SUV for malignant lesions in the neck region^36^; with the actual value being dependent on the anatomical location of the lesion and its proximity to bone structures.^36^,^37^ Thus, it seems reasonable that accounting for bone attenuation does practically not alter quantification of plaque tracer uptake at the location of the carotid bifurcation.

The major limitation of this study is the small number of data sets available, which hampers a reliable statistical evaluation of the data. Furthermore, two subjects with in total three plaque lesions were scanned twice. In this evaluation, the data from the repeated scans were assumed to be independent. However, this independence cannot be proven. The data originate from an oncological study, and not a dedicated vascular cohort, and no reference standard for the [^68^Ga]Ga-Pentixafor uptake in the evaluated plaque lesions was available due to the non-invasiveness of the plaque assessments. Thus, the reliable performance of the PVC method for ^68^Ga-tracers cannot be ensured. Therefore, the results of this study should be seen as an estimate of the influence of PVE and possible correction techniques.

## New Knowledge Gained

Quantitative measures of [68 Ga]Ga-Pentixafor uptake in carotid plaque lesions acquired and reconstructed using standard PET methods are unreliable due to the strong influence of PVEs. Uptake differences between differently classified plaque lesions may be seen in group wise comparisons. However, for the use of [68 Ga]Ga-Pentixafor for the classification of inflammatory processes in individual lesions, proper PVC is required.

## Conclusions

PVEs significantly influence quantitative readings of [68 Ga]Ga-pentixafor uptake in atherosclerotic plaques. SUV and TBR from standard [68 Ga]Ga-pentixafor PET images do not appear to be useful for the classification of atherosclerotic plaques in individual patients given the inherently, significant PVE. While, PVC helps to enhance the differences of [68 Ga]Ga-pentixafor uptake measurements across plaque types, absolute quantification of tracer concentrations in these lesions is still questionable. The effect of ignoring bone in standard MR-AC methods is clinically irrelevant for the assessment of atherosclerotic plaques located near the carotid bifurcation.

## Electronic supplementary material

Below is the link to the electronic supplementary material.

Supplementary material 1 (DOCX 19 kb)

Supplementary material 2 (PPTX 1208 kb)

## Abbreviations

AC: Attenuation correction
FWHM: Full-width at half-maximum
LP: Local projection
MALT: Mucosa-associated lymphoid tissue
PET: Positron emission tomography
PET/MRI: Positron emission tomography/magnetic resonance imaging
PVC: Partial volume correction
PVE: Partial volume effect
TBR: Tissue-to-background ratios
VOI: Volume-of-interest
TSE: Turbo spin echo
