# Supplementary material for: Accuracy of PET quantification in [68Ga]Ga-pentixafor PET/MR imaging of carotid plaques
Source: J Nucl Cardiol. 2020 Jul 21;29(2):492–502. doi: 10.1007/s12350-020-02257-3 (PMC8993720; doi:10.1007/s12350-020-02257-3)
Supplement: Supplementary file 1 — Supplementary material 1 (DOCX 19 kb) [file 12350_2020_2257_MOESM1_ESM.docx]

**Supplement**

**Accuracy of PET quantification in [^68^Ga]Ga-Pentixafor PET/MR imaging of carotid plaques**

**Supplement Table 1:**

Table summarizes size, plaque type and maximum (max) and mean TBR for standard (std) image reconstructions and PVC corrected PET. The LP_TBR_Prior value is the PVC prior used as a reconstruction prior within the LP PVC method. It can be described as the theoretically calculated activity value of the respective plaque.

| Plaque  ID | Size  [mm3] | Plaque  Type | TBR_std_max | TBR_std_mean | TBR_PVC_max | TBR_PVC_mean | LP_TBR_Prior |
| --- | --- | --- | --- | --- | --- | --- | --- |
| 1 | 77 | 1 | 1,483 | 1,251 | 1,064 | 0,931 | 0,708 |
| 2 | 100 | 1 | 1,910 | 1,133 | 1,527 | 0,924 | 0,846 |
| 3 | 159 | 2 | 1,277 | 1,152 | 1,637 | 1,280 | 1,719 |
| 4 | 284 | 2 | 1,598 | 1,326 | 2,567 | 1,817 | 2,562 |
| 5 | 154 | 2 | 1,638 | 1,460 | 1,762 | 1,528 | 1,889 |
| 6 | 91 | 2 | 1,678 | 1,318 | 2,701 | 1,994 | 3,806 |
| 7 | 75 | 2 | 1,719 | 1,416 | 3,010 | 2,199 | 4,317 |
| 8 | 37 | 2 | 1,050 | 1,017 | 2,318 | 2,000 | 2,621 |
| 9 | 79 | 2 | 1,733 | 1,506 | 2,096 | 1,931 | 2,632 |
| 10 | 38 | 2 | 1,121 | 1,107 | 6,243 | 4,514 | 7,812 |
| 11 | 168 | 2 | 1,462 | 1,352 | 3,897 | 3,682 | 4,165 |
| 12 | 31 | 2 | 1,627 | 1,550 | 2,138 | 1,822 | 2,876 |
| 13 | 143 | 2 | 1,683 | 1,512 | 4,534 | 3,150 | 4,739 |
| 14 | 33 | 2 | 0,758 | 0,625 | 2,476 | 1,501 | 2,788 |
| 15 | 218 | 3 | 1,538 | 1,279 | 3,172 | 2,383 | 3,249 |
| 16 | 175 | 3 | 1,687 | 1,381 | 2,600 | 2,035 | 2,579 |
| 17 | 168 | 3 | 1,640 | 1,376 | 6,912 | 5,750 | 7,401 |
| 18 | 202 | 3 | 1,595 | 1,311 | 2,094 | 1,718 | 2,237 |
| 19 | 146 | 3 | 1,310 | 1,178 | 3,955 | 2,468 | 3,517 |
| 20 | 138 | 3 | 1,066 | 1,012 | 2,219 | 1,841 | 2,422 |
| 21 | 97 | 3 | 1,393 | 1,289 | 2,391 | 2,060 | 2,870 |
| 22 | 143 | 3 | 1,263 | 0,881 | 2,407 | 2,009 | 2,652 |
| 23 | 119 | 3 | 1,361 | 1,237 | 2,753 | 2,097 | 2,930 |
| 24 | 52 | 3 | 1,221 | 1,155 | 1,733 | 1,480 | 1,962 |
| 25 | 94 | 3 | 2,200 | 1,577 | 2,629 | 2,260 | 2,631 |
| 26 | 127 | 3 | 1,647 | 1,502 | 2,607 | 1,986 | 2,748 |
| 27 | 47 | 3 | 1,389 | 1,066 | 2,089 | 1,708 | 2,821 |
| 28 | 234 | 4 | 1,578 | 1,227 | 3,670 | 2,874 | 3,959 |
| 29 | 105 | 4 | 2,245 | 2,020 | 11,809 | 9,424 | 12,616 |
| 30 | 255 | 5 | 1,558 | 1,364 | 1,644 | 1,429 | 1,628 |
| 31 | 423 | 5 | 1,749 | 1,504 | 1,838 | 1,605 | 1,884 |
| 32 | 451 | 5 | 1,154 | 0,970 | 1,536 | 1,267 | 1,451 |
| 33 | 335 | 5 | 1,275 | 1,113 | 1,293 | 1,169 | 1,235 |
| 34 | 227 | 5 | 1,817 | 1,377 | 1,537 | 1,224 | 1,016 |
