# Supplementary material for: Accuracy of PET quantification in [68Ga]Ga-pentixafor PET/MR imaging of carotid plaques
Source: J Nucl Cardiol. 2020 Jul 21;29(2):492–502. doi: 10.1007/s12350-020-02257-3 (PMC8993720; doi:10.1007/s12350-020-02257-3)
Supplement: Supplementary file 2 — Supplementary material 2 (PPTX 1208 kb) [file 12350_2020_2257_MOESM2_ESM.pptx]

## Slide 1
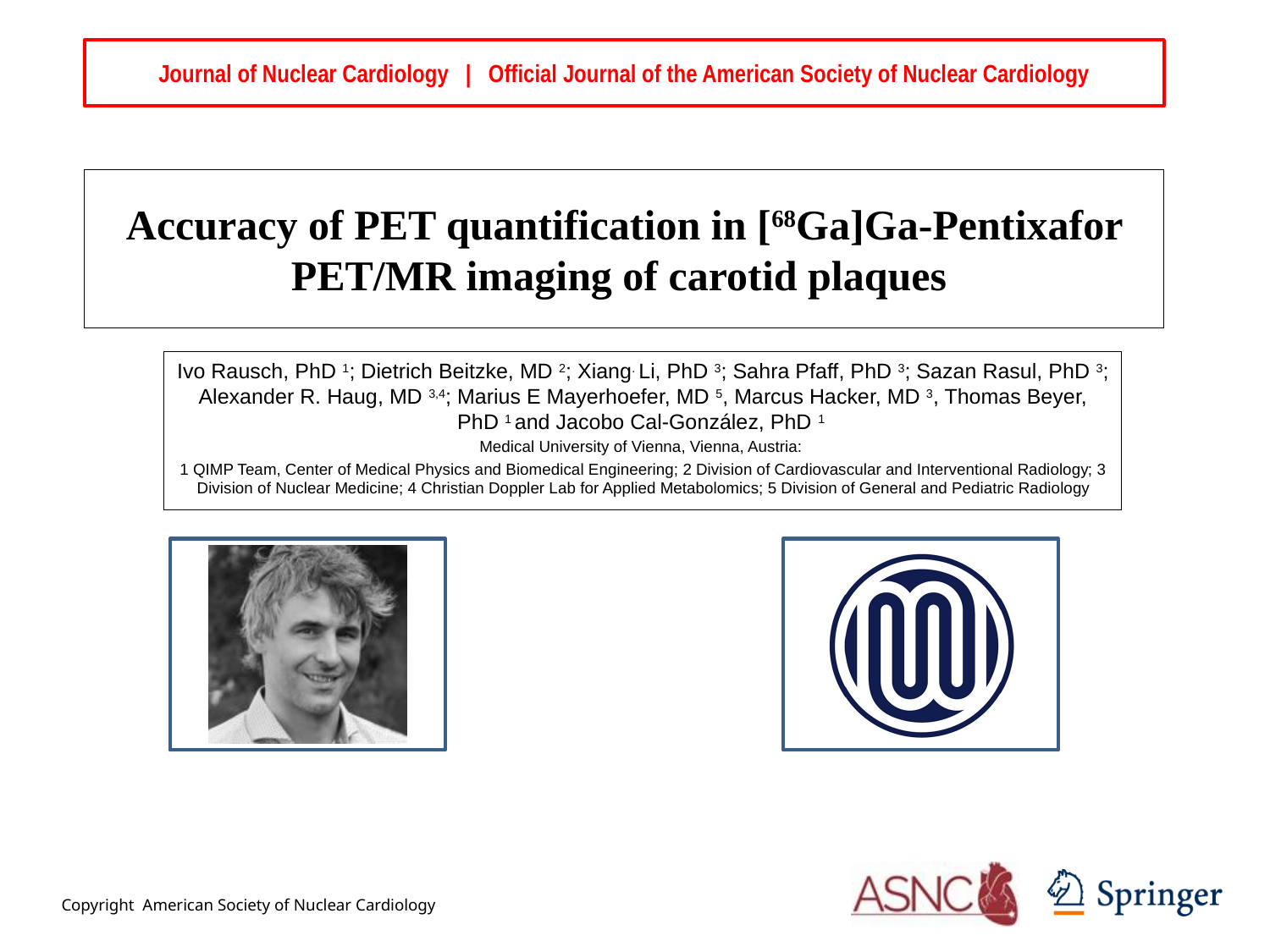

Journal of Nuclear Cardiology | Official Journal of the American Society of Nuclear Cardiology
# Accuracy of PET quantification in [68Ga]Ga-Pentixafor PET/MR imaging of carotid plaques
Ivo Rausch, PhD 1; Dietrich Beitzke, MD 2; Xiang. Li, PhD 3; Sahra Pfaff, PhD 3; Sazan Rasul, PhD 3; Alexander R. Haug, MD 3,4; Marius E Mayerhoefer, MD 5, Marcus Hacker, MD 3, Thomas Beyer, PhD 1 and Jacobo Cal-González, PhD 1
Medical University of Vienna, Vienna, Austria:
1 QIMP Team, Center of Medical Physics and Biomedical Engineering; 2 Division of Cardiovascular and Interventional Radiology; 3 Division of Nuclear Medicine; 4 Christian Doppler Lab for Applied Metabolomics; 5 Division of General and Pediatric Radiology
Head shot of author
required
Institution
Picture/Logo
Optional
Copyright American Society of Nuclear Cardiology

## Slide 2
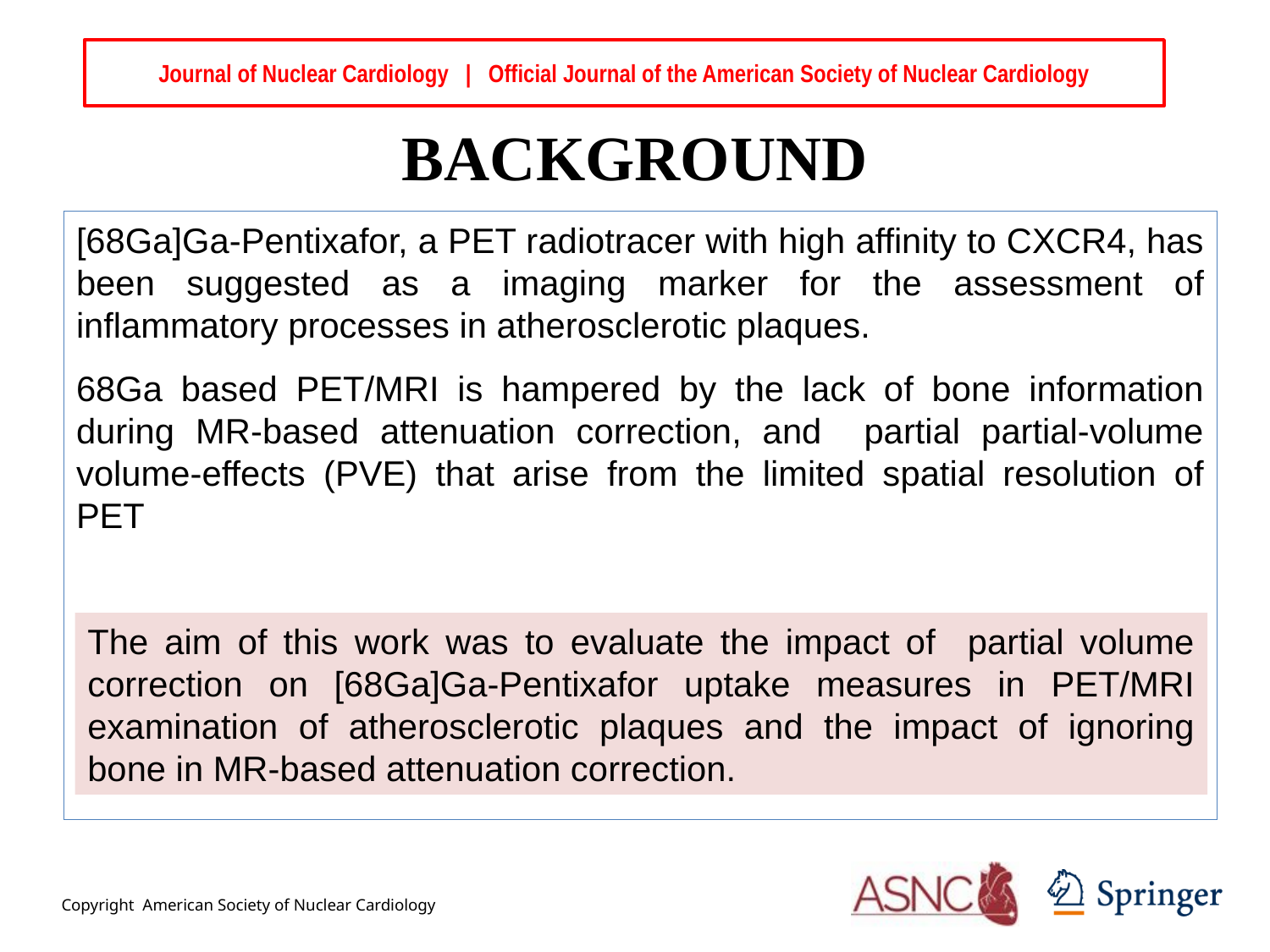

Journal of Nuclear Cardiology | Official Journal of the American Society of Nuclear Cardiology
# BACKGROUND
[68Ga]Ga-Pentixafor, a PET radiotracer with high affinity to CXCR4, has been suggested as a imaging marker for the assessment of inflammatory processes in atherosclerotic plaques.
68Ga based PET/MRI is hampered by the lack of bone information during MR-based attenuation correction, and partial partial-volume volume-effects (PVE) that arise from the limited spatial resolution of PET
The aim of this work was to evaluate the impact of partial volume correction on [68Ga]Ga-Pentixafor uptake measures in PET/MRI examination of atherosclerotic plaques and the impact of ignoring bone in MR-based attenuation correction.
The aim of this work was to evaluate the impact of partial volume correction on [68Ga]Ga-Pentixafor uptake measures in PET/MRI examination of atherosclerotic plaques and the impact of ignoring bone in MR-based attenuation correction.
Copyright American Society of Nuclear Cardiology

## Slide 3
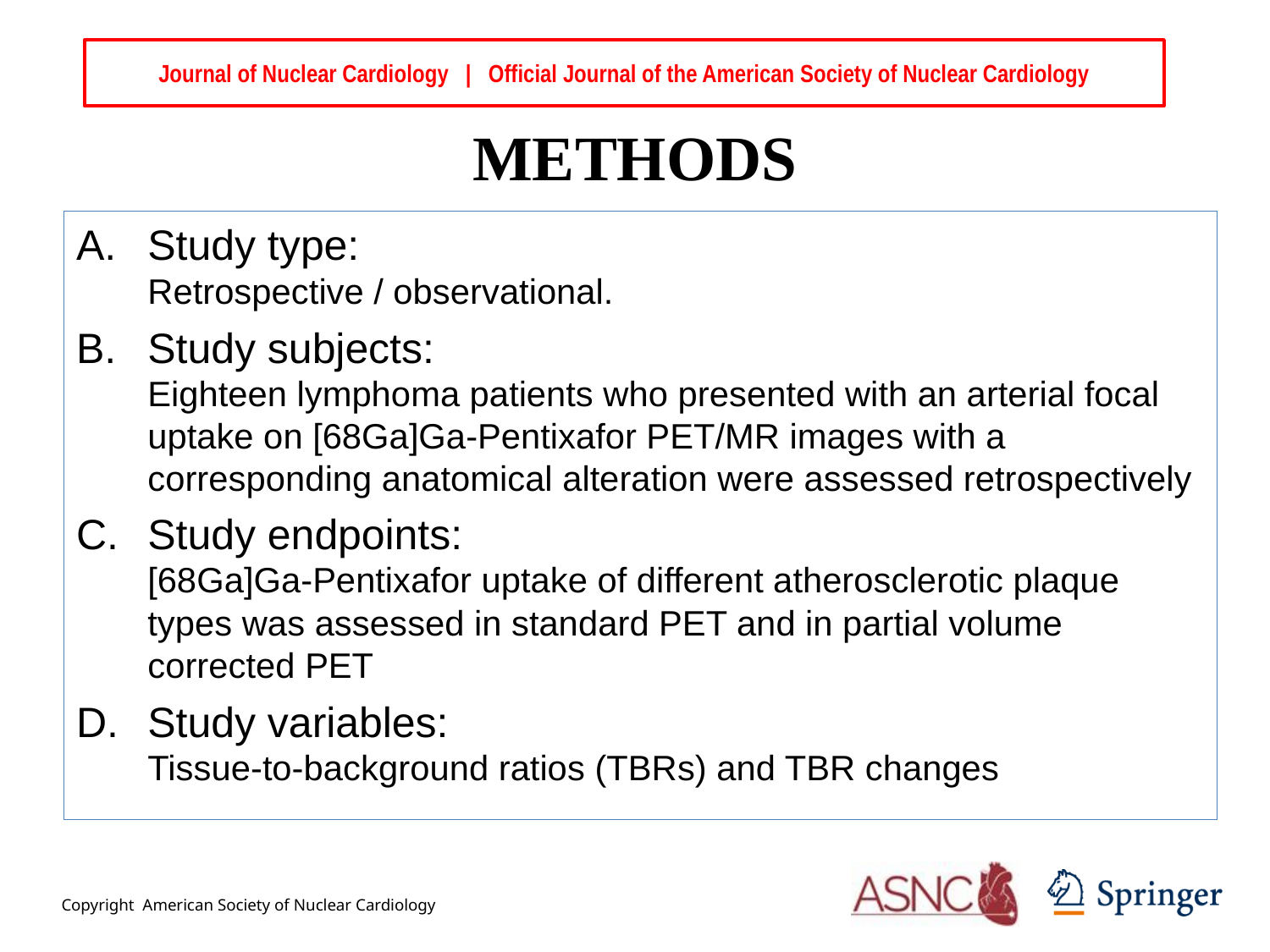

Journal of Nuclear Cardiology | Official Journal of the American Society of Nuclear Cardiology
# METHODS
Study type:
Retrospective / observational.
Study subjects:
Eighteen lymphoma patients who presented with an arterial focal uptake on [68Ga]Ga-Pentixafor PET/MR images with a corresponding anatomical alteration were assessed retrospectively
Study endpoints:
[68Ga]Ga-Pentixafor uptake of different atherosclerotic plaque types was assessed in standard PET and in partial volume corrected PET
Study variables:
Tissue-to-background ratios (TBRs) and TBR changes
Copyright American Society of Nuclear Cardiology

## Slide 4
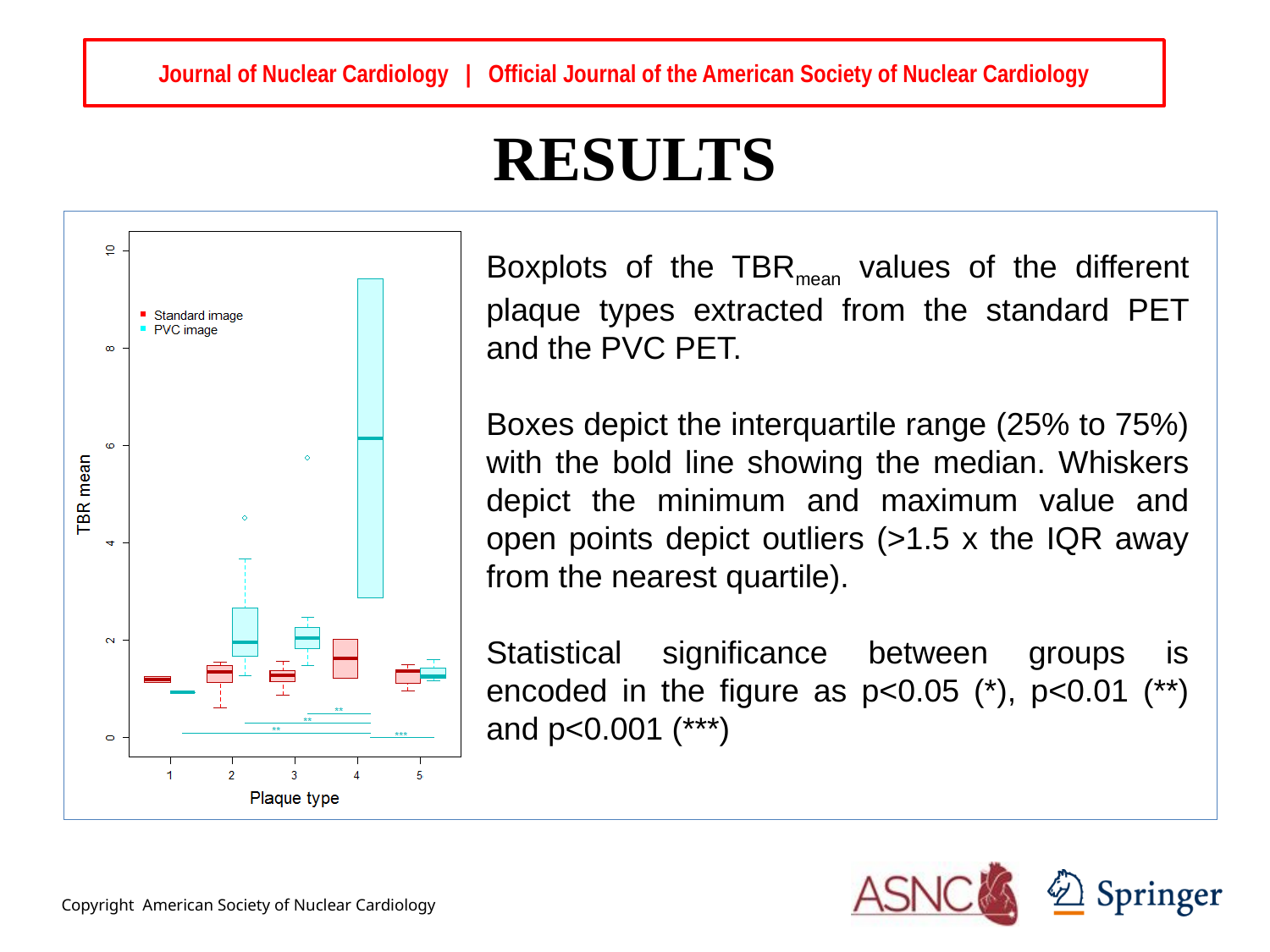

Journal of Nuclear Cardiology | Official Journal of the American Society of Nuclear Cardiology
# RESULTS
Boxplots of the TBRmean values of the different plaque types extracted from the standard PET and the PVC PET.
Boxes depict the interquartile range (25% to 75%) with the bold line showing the median. Whiskers depict the minimum and maximum value and open points depict outliers (>1.5 x the IQR away from the nearest quartile).
Statistical significance between groups is encoded in the figure as p<0.05 (*), p<0.01 (**) and p<0.001 (***)
Copyright American Society of Nuclear Cardiology

## Slide 5
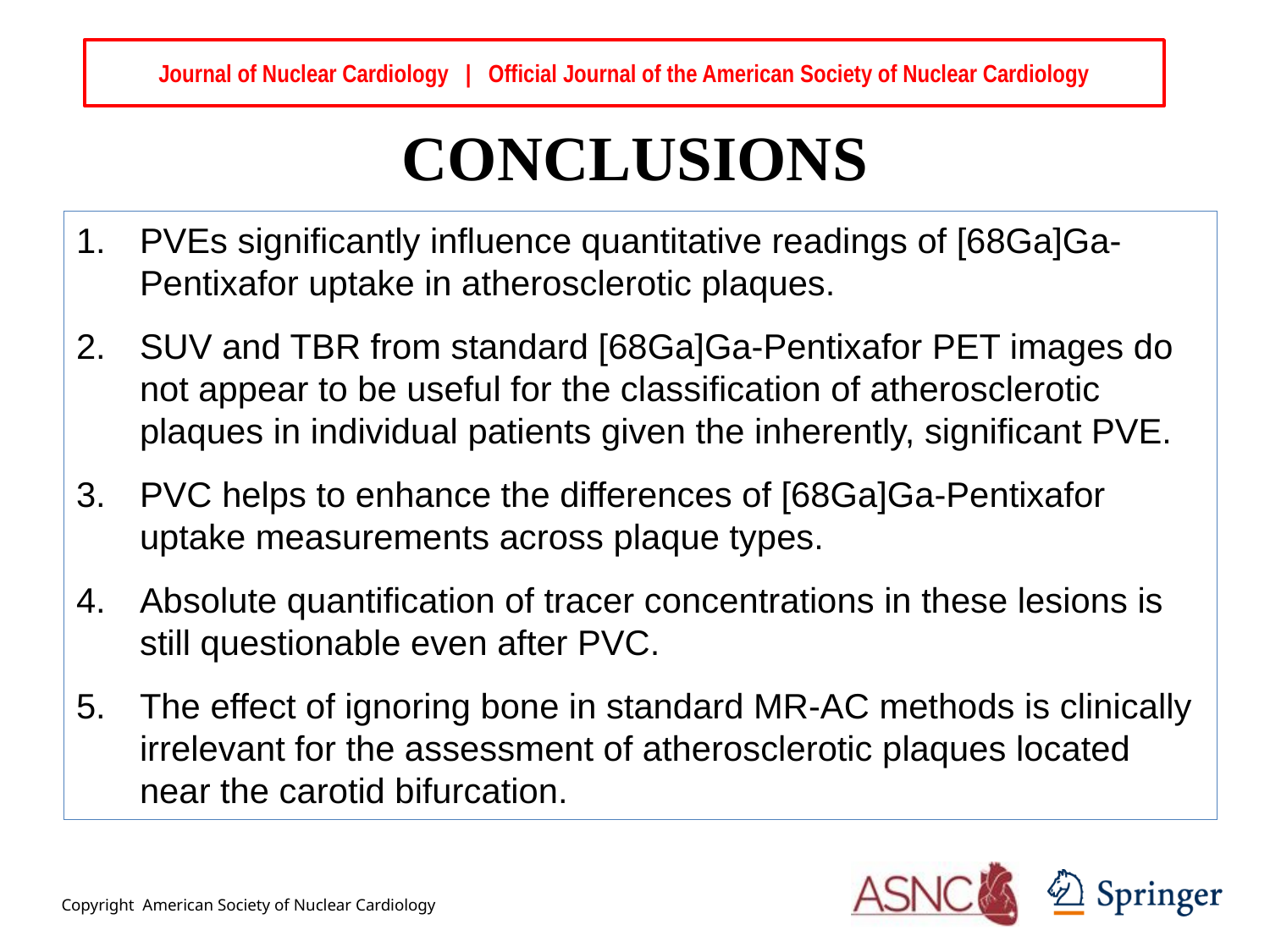

Journal of Nuclear Cardiology | Official Journal of the American Society of Nuclear Cardiology
# CONCLUSIONS
PVEs significantly influence quantitative readings of [68Ga]Ga-Pentixafor uptake in atherosclerotic plaques.
SUV and TBR from standard [68Ga]Ga-Pentixafor PET images do not appear to be useful for the classification of atherosclerotic plaques in individual patients given the inherently, significant PVE.
PVC helps to enhance the differences of [68Ga]Ga-Pentixafor uptake measurements across plaque types.
Absolute quantification of tracer concentrations in these lesions is still questionable even after PVC.
The effect of ignoring bone in standard MR-AC methods is clinically irrelevant for the assessment of atherosclerotic plaques located near the carotid bifurcation.
Copyright American Society of Nuclear Cardiology
